# Permanent Draft Genome Sequence of *Frankia* sp. Strain BR, a Nitrogen-Fixing Actinobacterium Isolated from the Root Nodules of *Casuarina equisetifolia*

**DOI:** 10.1128/genomeA.01000-16

**Published:** 2016-09-15

**Authors:** Timothy D’Angelo, Rediet Oshone, Feseha Abebe-Akele, Stephen Simpson, Krystalynne Morris, W. Kelley Thomas, Louis S. Tisa

**Affiliations:** University of New Hampshire, Durham, New Hampshire, USA

## Abstract

*Frankia* sp. strain BR is a member of *Frankia* lineage Ic and is able to reinfect plants of the *Casuarinaceae* family. Here, we report a 5.2-Mbp draft genome sequence with a G+C content of 70.0% and 4,777 candidate protein-encoding genes.

## GENOME ANNOUNCEMENT

Members of the genus *Frankia* are nitrogen-fixing actinobacteria that form an endophytic symbiosis with actinorhizal plants (1). This group of woody dicotyledonous plants is distributed worldwide and comprises over 200 species from eight angiosperm families (2). Because of its symbiosis with *Frankia* spp., actinorhizal plants play important ecological roles as pioneer species and are used in agroforestry, land reclamation, crop protection, and soil stabilization projects (3–5). Molecular phylogenetic approaches have identified four major clusters of *Frankia* (6–9), and genomes for representatives from each cluster have been sequenced (10–28). The availability of these *Frankia* genome databases has opened up the use of “omics” approaches. Furthermore, analysis of *Frankia* genomes has revealed new potential in respect to metabolic diversity, natural product biosynthesis, and stress tolerance, which may aid the cosmopolitan nature of the actinorhizal symbiosis.

Actinorhizal plants belonging to the *Casuarinaceae* family are able to grow under saline conditions and have been used extensively as a green barrier (28–33). *Casuarina* trees are native to tropical areas of Australia and the South Pacific and have subsequently been transplanted to other tropical areas of the world, particularly Africa (34). In countries such as Egypt, Tunisia, and Senegal, *Casuarina* trees are actively used in agroforestry and as windbreaks and shelterbelts for agriculture in arid areas (32, 35, 36). Members of *Frankia* cluster 1c have the most restricted host range and are able to infect only *Casuarina* and *Allocasuarina* host plants (1, 18). *Frankia* sp. strain BR was isolated from axenic *Casuarina equisetifolia* root nodules that had been inoculated with crushed *C. equisetifolia* root nodules from Brazil (37). The *Frankia* sp. strain BR genome was sequenced to provide information on cluster 1c lineage and symbiosis with *Casuarina* trees.

Sequencing of the draft genome of *Frankia* sp. strain BR was performed at the Hubbard Center for Genome Studies (University of New Hampshire, Durham, NH, USA) using Illumina technology techniques (38). A standard Illumina shotgun library was constructed and sequenced using the Illumina HiSeq2500 platform, which generated 24,720,192 reads (260-bp insert size) totaling 3,708 Mbp. The Illumina sequence data were assembled using CLC Genomics workbench version 8.5 and ALLPaths-LG version r41043 (39). The final draft assembly for *Frankia* sp. strain BR consisted of 180 contigs with an *N*_50_ contig size of 60.2 kb and 450.4× coverage of the genome. The final assembled genome contained a total sequence length of 5,227,240 bp with a G+C content of 70.0%.

The assembled *Frankia* sp. strain BR was annotated via the Integrated Microbial Genomes (IMG) platform developed by the Joint Genome Institute, Walnut Creek, CA, USA (40, 41), resulting in 4,777 candidate protein-encoding genes and 46 tRNA and two rRNA regions.

### Accession number(s).

This whole-genome shotgun sequence has been deposited at DDBJ/EMBL/GenBank under the accession number LRTJ00000000. The version described in this paper is the first version, LRTJ01000000.
